# Supplementary material for: Inflammation as a Prognostic Marker in Cardiovascular Kidney Metabolic Syndrome: A Systematic Review
Source: Int J Mol Sci. 2025 Dec 22;27(1):134. doi: 10.3390/ijms27010134 (PMC12785973; doi:10.3390/ijms27010134)
Supplement: Supplementary file 1 [file ijms-27-00134-s001.zip › ijms-3955052-supplementary.pdf]

Supplementary Data

Supplementary *S1* Search Strings

PubMed

((((((((Inflammation) OR ("Inflammatory measures")) OR ("Inflammatory indicators")) OR ("Inflammatory profiles")) OR ("Inflammatory scores")) OR ("Inflammatory parameters")) OR ("Inflammatory markers")) OR ("Inflammatory indices")) AND (("cardiovascular kidney metabolic syndrome" OR CKMS OR CKM) OR (cardio-renal-metabolic))-30 June 2025

Web of Science

((((((((Inflammation) OR ("Inflammatory measures")) OR ("Inflammatory indicators")) OR ("Inflammatory profiles")) OR ("Inflammatory scores")) OR ("Inflammatory parameters")) OR ("Inflammatory markers")) OR ("Inflammatory indices")) AND (("cardiovascular kidney metabolic syndrome" OR CKMS OR CKM) OR (cardio-renal-metabolic))-30 June 2025

CINAHL

((((((((Inflammation) OR ("Inflammatory measures")) OR ("Inflammatory indicators")) OR ("Inflammatory profiles")) OR ("Inflammatory scores")) OR ("Inflammatory parameters")) OR ("Inflammatory markers")) OR ("Inflammatory indices")) AND (("cardiovascular kidney metabolic syndrome" OR CKMS OR CKM) OR (cardio-renal-metabolic)) MEDLINE -- MeSH 2025 -30 June 2025

Scopus

Inflammation OR "Inflammatory measures" OR "Inflammatory indicators" OR "Inflammatory profiles" OR "Inflammatory scores" OR "Inflammatory parameters" OR "Inflammatory markers" OR "Inflammatory indices" AND "cardiovascular kidney metabolic syndrome" OR CKMS OR CKM OR cardio-renal-metabolic-30 June 2025

Embase

('inflammation'/exp OR inflammation:ti,ab OR 'inflammatory measures':ti,ab OR 'inflammatory indicators':ti,ab OR 'inflammatory profiles':ti,ab OR 'inflammatory scores':ti,ab OR 'inflammatory parameters':ti,ab OR 'inflammatory markers':ti,ab OR 'inflammatory

indices':ti,ab) AND ('cardiorenal metabolic syndrome'/exp OR 'cardiovascular kidney metabolic syndrome':ti,ab OR CKMS:ti,ab OR CKM:ti,ab OR 'cardio-renal-metabolic':ti,ab)-30 June 2025

The final search was run on 30 June 2025 for all databases. Filters were applied during database searches to restrict results to clinical studies involving human adults and to exclude reviews and other non-original research and Rayyean was used.

Supplementary *S2* Inflammation as a Prognostic Marker in Cardiovascular Kidney Metabolic Syndrome: A Systematic Review.

Table S1: Quality in Prognostic Studies (QUIPS) tool for Risk of Bias, traffic-light representation.

**Included studies**	**Study Participants**	**Study Attrition**	**Prognostic Factor Measurement**	**Outcome Measurement**	**Study Confounding**	**Statistical Analysis Report**
**Cross-sectional Studies**						
Zhao et al., 2025						
Cao et al., 2025						
Asija et al, 2025						
Song et al, 2025						
Yu et al, 2025						
**Cohort and Longitudinal Studies**						
Zhang et al., 2025						
Han et al., 2025						
Chen et al., 2025						

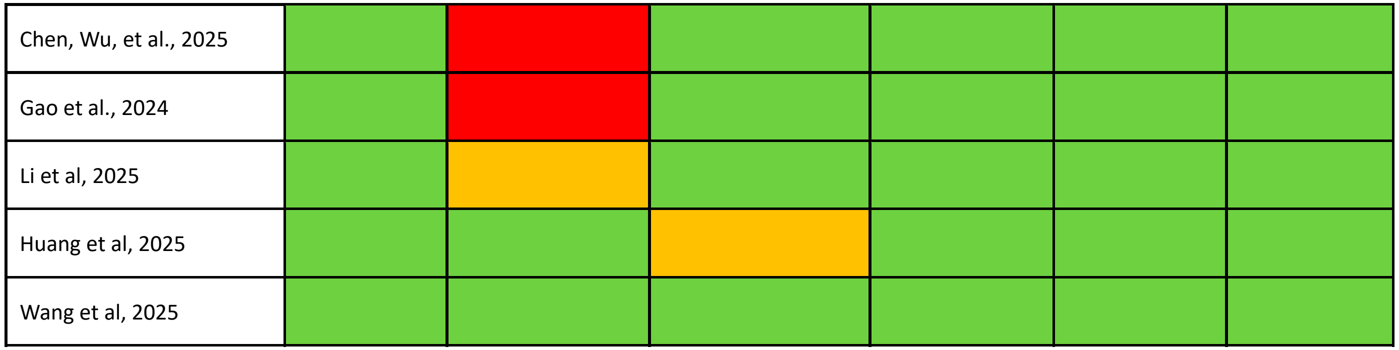

Key:

Low risk of bias   Moderate risk of bias   High risk of bias

Supplementary *S3*: Inflammation as a prognostic marker in Cardiovascular Kidney Metabolic Syndrome Systematic Review

Author(s): **Sihle E. Mabhida[1*], Haskly Mokoena[2], Mamakase G. Sello[3,4], Cindy George[1], Musawenkosi Ndlovu[1], Thabsile Mabi[1], Sisa Martins[1], Innocent S. Ndlovu[1], Onyemaechi Azu[3], André P. Kengne[1,5,6], Zandile J.R. Mchiza[1,3]**

Population: Adults (≥18 years) diagnosed with CKMS or its clinical components

Comparison: Individuals with lower levels or normal levels of inflammatory markers.

Assessment: Inflammatory indicators or indices (SIRI, SII, hs-CRP/HDL-C ratio, DII, TyG indices).

Outcome: Prognostic endpoints including all-cause mortality, cardiovascular mortality, CKMS progression, or composite adverse clinical outcomes

Certainty assessment							Impact	Certainty	Importance
№ of studies	Study design	Risk of bias	Inconsistency	Indirectness	Imprecision	Other considerations			
**Mortality Prediction (assessed with: Inflammatory Indices used: TyG, hs-CRP/HDL-C ,SIRI, SII+OBS ,CRP-TG)**									
6	non-randomised studies	not serious	not serious	serious[a]	not serious	none	Higher values of several inflammatory indices consistently predict increased mortality in CKMS populations. The global clinical impact remains uncertain due to population limits and outcome variability; indices warrant further validation.	⨁⨁⨁◯ Low Moderate[a]	CRITICAL
**Comorbidity Prediction (assessed with: Inflammatory Index used: SIRI)**									
1	non-randomised studies	not serious	not serious	serious[b,c]	not serious	none	Strong inflammatory signature (high SIRI) identifies patients at dramatically elevated risk for overlapping CKMS comorbidities, but evidence is limited, and findings may not extrapolate to other settings or health systems.	⨁⨁⨁◯ Low Moderate[b,c]	CRITICAL
**CKMS Risk / Prevalence (assessed with: Inflammatory Indices used : E-DII, SII, NHR/LHR/MHR,RAR, NPAR, SIRI, HOMA-IR)**									
5	non-randomised studies	not serious	not serious	not serious	not serious	all plausible residual confounding would reduce the demonstrated effect	Multiple inflammatory indices are associated with modestly increased risk and prevalence of CKMS, but certainty is low, and global generalizability is limited. Use for risk stratification should be cautious outside high-resource settings.	⨁⨁⨁◯ Low Moderate	CRITICAL
**Mental Health–Inflammation pathway (assessed with: PHQ-9 score, SIRI mediation)**									
1	non-randomised studies	not serious	not serious	serious[d]	not serious	none	Inflammatory burden (SIRI) partly explains the link between depressive symptoms and increased mortality among those with CKMS, but evidence is preliminary, and applicability to other populations and cultures is uncertain.	⨁⨁⨁◯ Low Moderate[d]	IMPORTANT

CI: confidence interval

Explanations

a. Mortality associations were drawn mainly from US and Chinese cohorts

b. SIRI-based multi-morbidity prediction was evaluated only in one US population sample (NHANES).

c. The prevalence and clustering of comorbidities (heart failure, CKD, CAD) are shaped by region-specific factors and may not generalize to populations with different disease burden or healthcare systems.

d. All findings were based on US cohorts, where mental health reporting, management, and the epidemiologic linkage to inflammation may diverge from other populations.

Supplementary *S4* CKMS extraction form

Authors	Country	Study design	Population	Avarage age	CKM Stage	Inflammatory Indices
(Cao et al., 2025)	USA	Prospective	29,459	49.8	0-4	SIRI
(Chen, Lian, et al., 2025)	USA	Prospective	18,295	45.2	0-3	SIRI
(Zhang et al., 2025)	USA	Prospective	6383	49.9	0-3	TyG-WC
(Han et al., 2025)	China	Prospective	6719	59.0	1-4	hs-CRP/HDL-C
(Li et al., 2025)	China	Prospective	17,705	Mid-to-late-adulthood	0-3	CRP–TG Index (CTI)
(Wang et al., 2025)	USA	Prospective	12,314	Adults	1-4	PHQ-9 score, SIRI mediation
(Huang et al., 2025)	USA	Retrospective	19,884	Adults	0-4	RAR, NPAR, SIRI, HOMA-IR
(Chen, Wu, et al., 2025)	USA	Prospective	21,609	52.0	1-4	SII

Follow-up duration	Outcome measure	Related to all mortality (HR [CI])	Related to CVDs mortality (HR [CI])
109 months	Associated with higher risks of all-cause and CVD mortality	1.84 [1.65-2.05]	2.5 [2-3.12]
121 months	Independent risk factors for mortality	1.16 [1.11-1.21]	1.33 [1.19-1.46]
10 years	All-cause mortality and cardiovascular mortality	1.5 [1.18-1.92]	1.85 [1.19-2.86]
10 years	CRP/HDL-C ratio and long-term mortality risk	1.15 [1.09-1.22]	Not reported

2 years	Linear association between CTI and all-cause death	1.95 [1.28-2.97]	1.52 [1.44-1.59]
6.75 years	Correlated with increased all-cause mortality	1.07 [0.69-1.64]	Not reported
**Not reported**	Significant relationship between RAR, NPAR, SIRI, and Homair with the five stages of CKM	2.38 [1.98-2.88]	Not reported
9.3 years	Increase in all cause of mortality	1.18 [1.06-1.32]	1.26 [1.01-1.57]

Supplementary *S5* Meta analysis

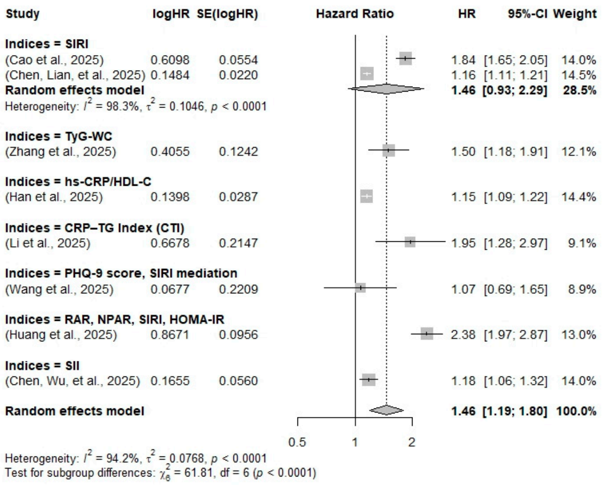

Supplementary *S6* PROSPERO URL

https://www.crd.york.ac.uk/PROSPERO/view/CRD420251131929.

Supplementary *S7*. Characteristic key findings on the included cross-sectional studies.

**Ref.**	**Study Design & Setting**	**Population (N, Mean Age, Gender)**	**Inflammatory Indices**	**Key findings**
Zhao et al., 2025 [22]	Cross-sectional, NHANES (USA)	24 071 participants; mean age 43.9 years; 49.3% female	E-DII	Each unit increase in the E-DII was associated with a 12% higher likelihood of CKMS; the association was strongest among women aged 40 years and older and among participants who self-identified as non-Hispanic White.
Gao et al., 2024 [28]	Cross-sectional, China	91 351 participants; mean age 42.8 years; 56% female	SII	A higher Systemic SII showed a dose-dependent, non-linear association with the likelihood of CKMS and a linear association with the risk of CKD.
Asija et al, 2025 [27]	Cross-sectional, NHANES (USA)	7 582 participants; median Systemic Inflammation Response Index 0.94	SIRI	A SIRI >0.94 was strongly associated with the presence of combined heart failure, CKMS, and related comorbidities; for example, the odds of simultaneously having heart failure, coronary artery disease, and CKD were approximately fourteen times higher in this group.
Song et al, 2025 [26]	Cross-sectional, NHANES (USA)	19 534 participants (weighted)	NHR, LHR, MHR	Participants in the highest tertile for each ratio had higher odds of stages one to four of CKMS, displaying a clear dose-response pattern.

Yu et al, 2025 [25]	Cross-sectional, NHANES (USA)	7 110 adults aged twenty to seventy-nine years	E-DII	A linear increase in the E-DII was associated with a higher prevalence of CKMS; alcohol intake contributed most strongly to the inflammatory index.
